# The Effect of Mild Exercise in the Chemotherapy Room on the Anxiety Level of Cancer Patients: A Prospective Observational Paired Cohort Study

**DOI:** 10.3390/jcm14155591

**Published:** 2025-08-07

**Authors:** Christina Mavrogiannopoulou, Georgios Papastratigakis, Emmanouela Koutoulaki, Panagiotis Vardakis, Georgios Stefanakis, Athanasios Kourtsilidis, Kostantinos Lasithiotakis, Alexandra Papaioannou, Vasileia Nyktari

**Affiliations:** 1 School of Medicine, University of Crete, 71003 Heraklion, Greece; x.mavrogiannopoulou@gmail.com (C.M.); papastratigakisg@gmail.com (G.P.); emmanouelakt@yahoo.gr (E.K.); athanasioskourts@gmail.com (A.K.); k.lasithiotakis@uoc.gr (K.L.); papaioaa@uoc.gr (A.P.); 2 Department of Anaesthesiology, University Hospital of Heraklion, 71110 Crete, Greece; vardpan178@gmail.com; 3 Department of Anaesthesiology, Children’s Hospital “Paidwn Agia Sofia”, 11527 Athens, Greece; 4 Department of Medicine, University of Thessaly, 41500 Larissa, Greece; gstefanakis@uth.gr

**Keywords:** exercise therapies, chemotherapy, anxiety

## Abstract

**Background/Objectives:** Cancer represents a significant health challenge, with high mortality and morbidity rates. Its diagnosis often triggers chronic stress, adversely affecting patient outcomes. Exercise has emerged as complementary therapy, enhancing treatment adherence and mitigating the side effects of chemotherapy. This study examines the effects of mild exercise during chemotherapy on patient anxiety. **Methods:** This prospective paired cohort study was conducted in the General Oncology Hospital of Kifisia “Agioi Anargyroi” in Athens, Greece. Adult cancer patients undergoing chemotherapy participated, excluding those with cognitive, hearing, or motor impairments, those who experienced side effects, or those who declined consent. Anxiety was measured before and after a 20-minute exercise routine performed during chemotherapy, using the Greek-translated State–Trait Anxiety Inventory (STAI). The exercise regimen included warm-up, full-body stretching, and cool-down exercises. Pre- and post-exercise scores were analyzed using the Wilcoxon signed-rank test. **Results:** Forty-five patients (20 women, 25 men; mean age 69.02 ± 10.62 years) with various cancer backgrounds participated. Pre-intervention anxiety levels were in the borderline “moderate” range, dropping post-exercise to the “low” range. Mean STAI scores decreased from 37.73 ± 13.33 to 32.00 ± 14.22 (*p* < 0.0001), with a medium-large effect size (Cohen’s d for paired samples = −0.646). No significant correlation was found between age and anxiety scores. **Discussion:** This study found a significant short-term reduction in anxiety, suggesting that incorporating mild exercise during chemotherapy may help in alleviating patient stress. The medium-to-large effect size supports the potential for meaningful short-term benefits. **Conclusions:** Incorporating mild exercise during chemotherapy may help reduce anxiety and psychological burden. These findings underscore the need for more comprehensive research in larger, more diverse populations to better understand the benefits of incorporating mild exercise during chemotherapy.

## 1. Introduction

In developed countries, it is estimated that approximately 20% of the population will be diagnosed with cancer at some point in their lives [1]. According to the Global Cancer Observatory, in 2022, lung cancer had the highest incidence among all cancer types, followed by breast, colorectal and prostate cancers [2]. The estimated numbers of new invasive cancer cases in the United States in 2025, ranked from higher to lower incidence, are mostly related to breast, prostate, lung and colorectal cancers [2]. Advancements in early diagnosis and treatment modalities have led to a significant improvement in survival rates, with some studies indicating an increase of up to 70%, thereby reclassifying cancer as a chronic disease in numerous cases [3,4]. Nonetheless, throughout the course of the disease and its associated treatment, patients frequently experience a range of physical symptoms—including pain, nausea, vomiting, and motor disabilities—as well as emotional distress, all of which can profoundly impact their health-related quality of life [QoL] [1,4].

A significant life event can increase the risk of psychological distress and anxiety [5,6]. Anxiety is a normal, potentially adaptive reaction in situations perceived as threatening, but becomes pathological when it is persistent and its severity or duration exceed normal expectations [7,8]. A cancer diagnosis, due to its inherently threatening nature, understandably elicits considerable anxiety among patients who must confront this existential threat. However, this anxiety may escalate into a clinically significant condition requiring therapeutic intervention. Anxiety has been recognized as a critical factor influencing QOL among cancer patients [7]. Furthermore, elevated levels of anxiety may exacerbate the side effects associated with chemotherapy, thereby further compromising QOL.

Risk factors for anxiety in cancer populations include several clinical and psychosocial variables, with chemotherapy emerging as a particularly prominent contributor [9]. Advanced disease stage and prolonged illness duration are associated with increased anxiety. Other risk factors include unemployment, younger age, and a higher prevalence of physical symptoms. Impairments in social and cognitive functioning, alongside insecure attachment styles, may further enhance anxiety. Moreover, poor communication with healthcare providers can intensify feelings of uncertainty and fear, contributing to increased anxiety [10]. A thorough understanding of these risk factors—especially those related to chemotherapy—is crucial for the development of targeted interventions in order to support patients throughout their cancer care journey.

Exercise is a valuable adjunct therapy for cancer patients, as it can mitigate many side effects and symptoms associated with both the disease and its treatment [11]. Research indicates that exercise significantly reduces cancer-related fatigue and psychosocial distress; it also enhances aerobic fitness, improves body composition and strength, and positively impacts various aspects of physical function and health-related QOL [12,13,14,15,16]. Moreover, regular physical activity may enhance relative dose intensity [RDI] by reducing chemotherapy-related toxicities, aiding recovery and overall health [7].

Current evidence regarding the benefits of exercise for cancer patients primarily focuses on exercise interventions conducted either during active treatment— excluding chemotherapy or radiotherapy—or after the completion of treatment. However, recent discussions have emphasized the potential value of engaging in physical exercise concurrently with chemotherapy infusions as a promising approach [17,18]. The aim of this prospective cohort study is to investigate the effects of mild exercise and stretching during chemotherapy on patients’ anxiety levels.

## 2. Materials and Methods

This prospective observational cohort study has received ethical approval from the Ethics Committee of the General Oncology Hospital of Kifissia “Agioi Anargyroi” in Athens, Greece [protocol number 779/15-1-2024]. The study was registered on ClinicalTrials.gov [Registration Number: NCT06943638]. It was conducted from 12 February 2024 to 24 March 2025, in the chemotherapy unit of “Agioi Anargyroi” General Oncology Hospital, in Athens.

The study population consisted of adult cancer patients aged ≥ 18 years undergoing chemotherapy. Prior to enrollment in the study, all participants provided informed consent after receiving a detailed printed guide outlining the nature of the intervention and their rights as research subjects. Patients were excluded if they declined participation, had cognitive or hearing impairments that could hinder their ability to participate, or if they were unable to mobilize independently. Participants were eligible regardless of their chemotherapy session number, and each participant was exposed to the intervention only once.

Sample size calculations, based on similar studies [17], determined a minimum of 45 participants to achieve adequate power [α = 0.01, β = 80%].

### 2.1. Intervention

The intervention was systematically designed and implemented by a licensed physiotherapist. Each session lasted 20 min and included mild exercises and a stretching routine tailored to meet the individual capabilities of each participant. The exercise plan was developed and tailored with careful consideration of hospital constraints and the specific medical conditions of each patient.

The design of the exercise protocol was based on the “Guidelines for implementing exercise programs for cancer patients” [19].

To address the concerns about the risk of injuries that many cancer patients and their families experience, we developed a program of low-intensity exercises. Patients received this exercise intervention only once, rather than throughout all their chemotherapy sessions.

Cancer treatments such as chemotherapy and radiation can lead to muscle and joint stiffness, which can limit the range of motion and make everyday activities challenging [20]. These treatments may also impact balance and coordination, making patients feel unsteady and increasing the risk of falls [21]. To address these issues, we have designed an exercise session focusing on flexibility and balance exercises. This exercise protocol aims to reduce tightness, enhance mobility, and improve balance, helping patients feel more comfortable and in control of their bodies.

**Figure 1:**
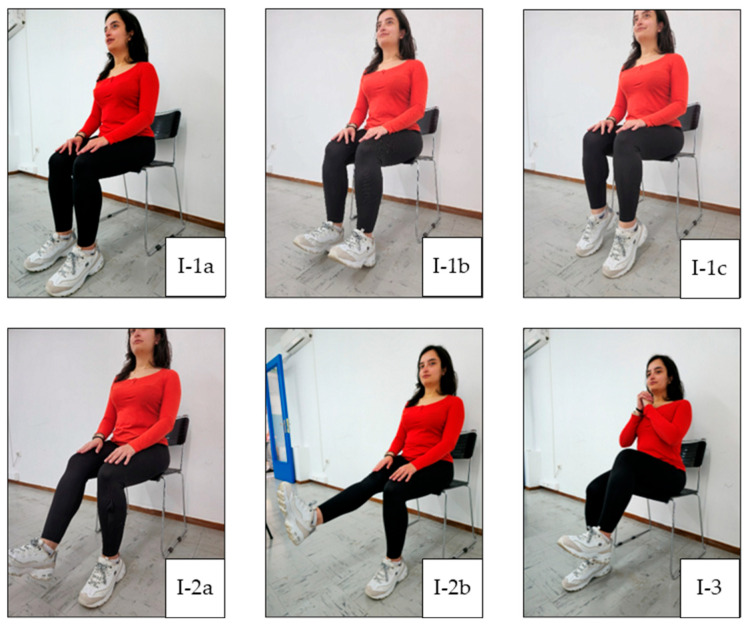
Warm up exercise. (**I-1a**) Starting position; (**I-1b**) dorsiflexion; (**I-1c**) plantar flexion; (**I-2a**) mid-range knee extension; (**I-2b**) full knee extension and isometric contraction; (**I-3**) knee lift.

**Figure 2:**
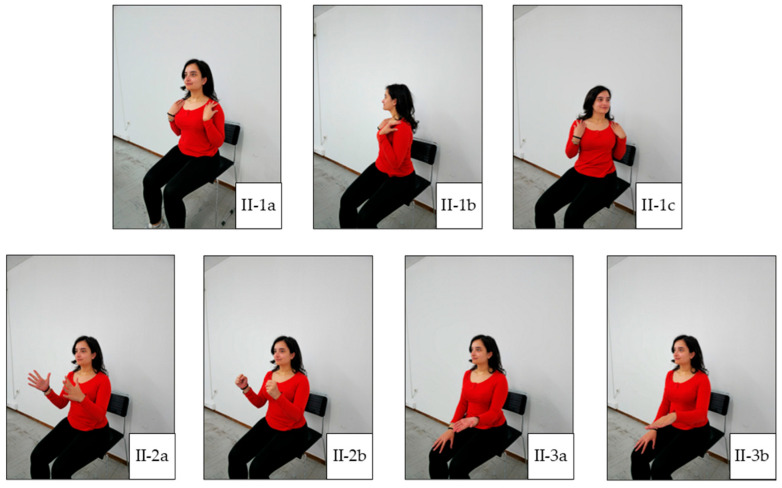
Main Exercise 1. (**II-1a**) Starting position for torso rotation; (**II-1b**) right rotation; (**II-1c**) left rotation; (**II-2a**) starting position for hand gripping; (**II-2b**) final position of hand gripping; (**II-3a**) forearm supination; (**II-3b**) forearm pronation.

**Figure 3:**
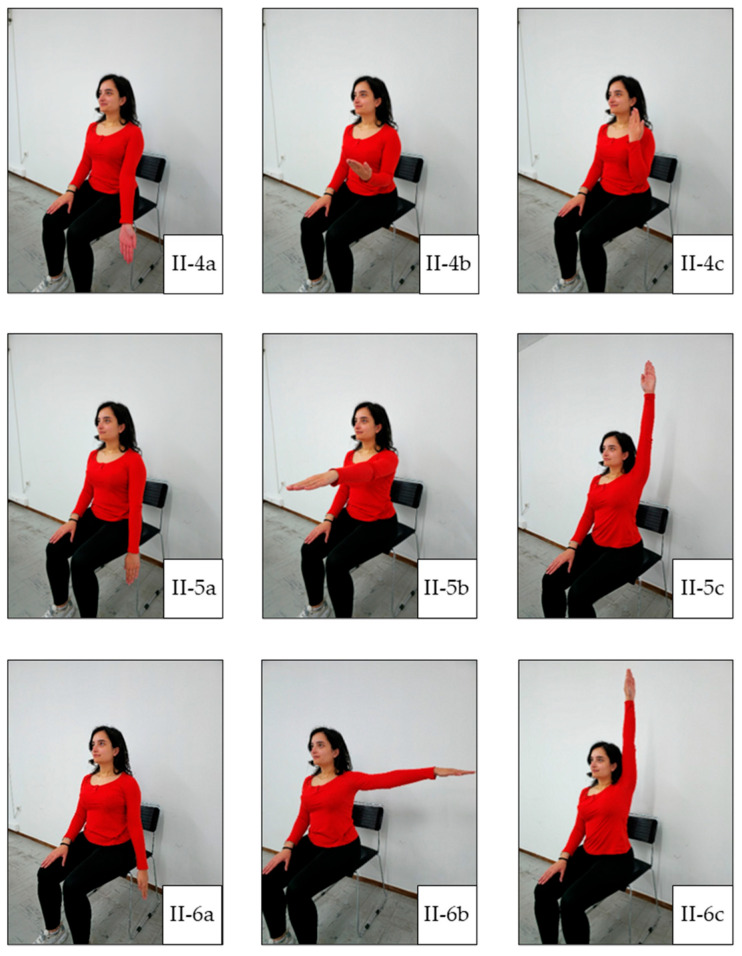
Main Exercise 2. (**II-4a**) Starting position of elbow flexion; (**II-4b**) mid-range of elbow flexion; (**II-4c**) end-range of elbow flexion; (**II-5a**) starting position of shoulder flexion; (**II-5b**) mid-position of shoulder flexion; (**II-5c**) final position of shoulder flexion; (**II-6a**) starting position of shoulder abduction; (**II-6b**) mid-position of shoulder abduction; (**II-6c**) final position of shoulder abduction.

**Figure 4:**
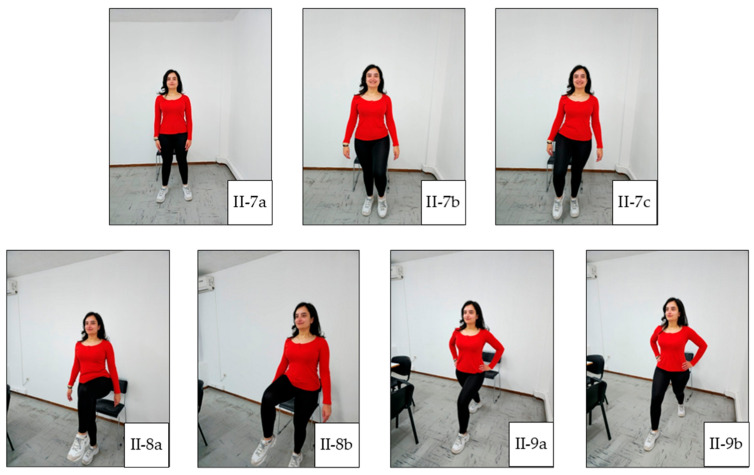
Main Exercise 3. (**II-7a**) Starting position; (**II-7b**) left small on-the-spot step; (**II-7c**) right small on-the-spot step; (**II-8a**) left high-knee step; (**II-8b**) right high-knee step; (**II-9a**) left leg lunge; (**II-9b**) right leg lunge.

**Figure 5:**
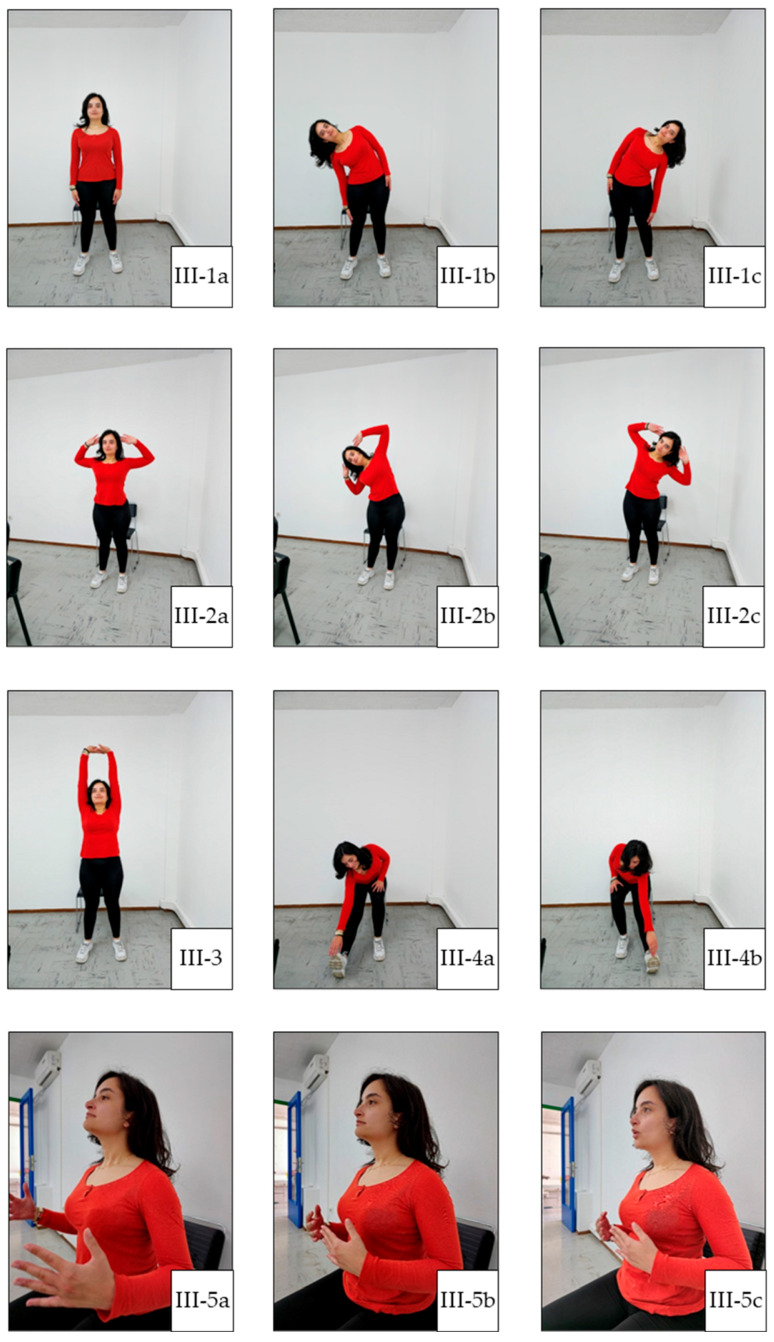
Cool-Down. (**III-1a**) Starting position for torso bend; (**III-1b**) right torso bend; (**III-1c**) left torso bend; (**III-2a**) starting position for standing lateral torso bends with arms raised; (**III-2b**) right standing lateral bend with arms raised; (**III-2c**) left standing lateral bend with arms raised; (**III-3**) overhead arm stretch; (**III-4a**) right seated hamstring stretch; (**III-4b**) left seated hamstring stretch; (**III-5a**) starting position for deep breath; (**III-5b**) deep inhale; (**III-5c**) exhale through the mouth.

In general, each exercise session consists of three main parts: warm-up, main training, and cool-down. Warm-up facilitates the transition from rest to exercise and may reduce the susceptibility to musculoskeletal injury by improving joint range of motion [22]. This prepares the body for further activity without sudden changes. A warm-up can include 5 to 10 min of low-intensity aerobic and cardio exercises, along with light stretching. The main training portion is when the primary exercise is performed. This can include a variety of activities, such as aerobic and cardio exercise, strength and resistance training, flexibility exercise, and balance exercise. Balance exercise is particularly beneficial for individuals with weakened bones or peripheral neuropathy, as it can help improve stability. The cool-down phase allows the heart rate and blood pressure to gently return to normal. It also aids in recovery and reduces post-exercise muscle soreness. The cool-down phase typically includes 5 to 10 min of relaxed, low-intensity activities, such as slow walking or cycling, as well as light stretching. Our exercise plan is illustrated in Figure 1, Figure 2, Figure 3, Figure 4 and Figure 5, and the full program is detailed in Table 1.

Prescribing the appropriate intensity of exercise can be challenging. In our session, all activities were characterized by light intensity, which is regarded as mild exercise. To explain exercise intensity practically to patients, we used the talk test, which is an effective tool for monitoring effort, ensuring that physical activity remains within a safe and beneficial range. During moderate exercise, a person can comfortably hold a conversation; when the effort level allows a person to talk and sing easily, it is classified as mild exercise. Conversely, if a participant can barely speak a few words before needing to catch their breath, they are probably exercising at a vigorous intensity [23]. After every few repetitions, participants were asked how they felt and needed to provide a response. If any participant had been unable to answer (which did not occur), we would have reduced the intensity of the exercise. Additionally, subjective effort was assessed through casual conversation, corresponding to ratings of 9–11 on the original Borg RPE scale (very light to light exertion) [24]. No physiological measures such as heart rate or blood pressure were taken; however, these observational and self-reported methods ensured that the activity remained within a safe and low-intensity range.

We used the Spielberger State–Trait Anxiety Inventory [STAI], which is a psychological inventory consisting of 40 self-report items on a 4-point Likert scale [25]. The STAI measures two types of anxiety—state anxiety [STAI-Y1] and trait anxiety [STAI-Y2]. For the purposes of this study, the STAI-State scale [Form Y-1] was employed, which specifically measures the levels of anxiety experienced at a given moment, referred to as “state anxiety”. This contrasts with “trait anxiety”, which reflects an individual’s general tendency to experience anxiety as a personality characteristic. We utilized the Greek-translated version of the STAI, validated by Liakos and Giannitsis in 1984 [26].

The Y-1 form consists of 20 statements. For each statement, the subject must choose one of the 4 alternatives to indicate how they feel: absolutely not, a little, enough, and very much. The scoring for the Y-1 form of the STAI-State scale ranges from 20 to 80, with higher scores indicating greater anxiety levels. Participants completed the STAI questionnaire in the chemotherapy room immediately after the start of chemotherapy treatment. Following this, the patient completed the exercise, and the anxiety assessment was performed again right afterward. Each participant served as their own control, allowing for a rigorous assessment of anxiety levels before and after the intervention. This design facilitates a thorough evaluation of the intervention’s effectiveness in alleviating anxiety.

### 2.2. Statistical Analysis

Due to the non-normal data distribution and paired nature of the measurements, the Wilcoxon signed-rank test was employed. Data differences Δ[After-Before] were normally distributed, and thus Cohen’s D for paired samples was used to determine effect size. Spearman’s rho was used to investigate possible correlations between gender, age, and the STAI scores, as well as their difference Δ[After-Before].

## 3. Results

**Figure 6:**
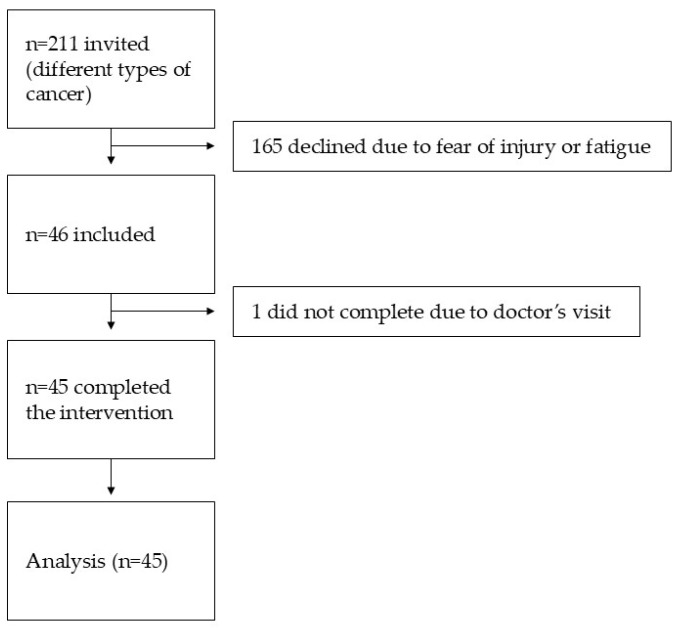
Patient recruitment flow chart. Notably, most eligible patients refused participation due to fear of injury or fatigue.

The study included 45 patients [20 women, 25 men] aged 53–85 years [mean ± SD: 69.02 ± 10.62] with various cancer types [e.g., hematological malignancies, ovarian cancer]. Patient recruitment is presented in Figure 6 as a flowchart. Of the 211 patients initially invited to participate, 165 declined due to concerns regarding potential injury or fatigue.

**Figure 7:**
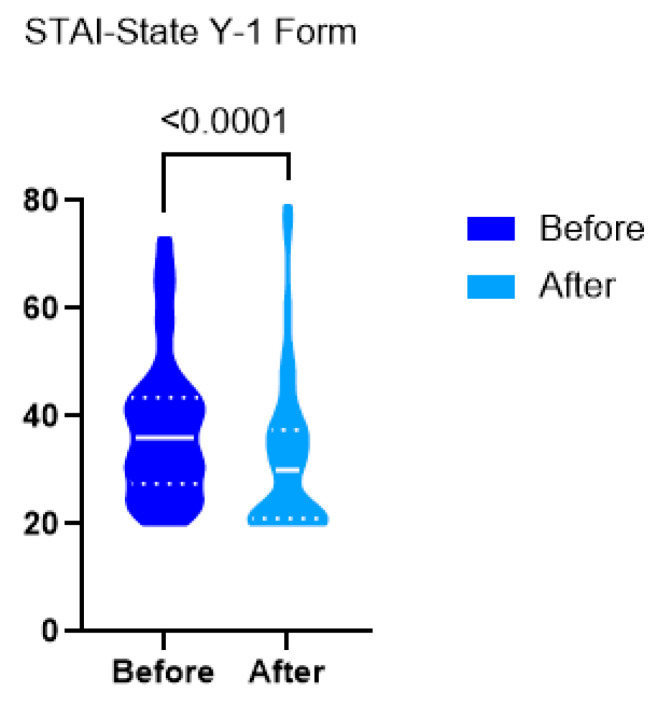
Y-1 Form of STAI-State questionnaire answers before and after exercise. The median is symbolized by a continuous horizontal line, quartiles by dotted horizontal lines.

The most pronounced improvement was observed in an 83-year-old woman, whose anxiety score decreased by 25 points [from 46 to 21]. Conversely, some patients, such as a 61-year-old woman with high initial anxiety, reported an increase [from 64 to 79]. Patients’ demographic data, as well as their pre- and post-exercise STAI-State scores, are presented in Table S1 of the Supplementary Material. The mean anxiety score decreased significantly from 37.73 ± 13.33 to 32.00 ± 14.22, with a *p*-value < 0.0001. Cohen’s D for paired samples was calculated to be d= −0.646, indicating a medium-large effect size. Anxiety scores before and after the intervention are summarized in Figure 7.

**Figure 8:**
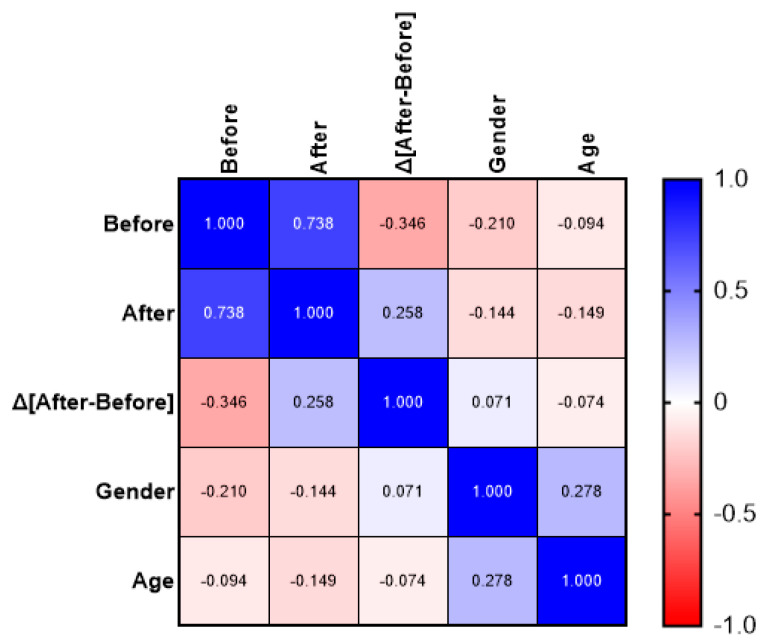
Spearman’s rho correlation analysis. Cells show Spearman’s rho values, representing the strength and direction of a monotonic association between variables (−1 to +1). Blue = positive correlation, red = negative correlation, white = no correlation; color intensity reflects strength of association.

Spearman’s rho did not reveal any significant correlations between age, gender, and the STAI scale values. The only significant correlation was a strong positive correlation between the before and after anxiety values [rho = 0.738, *p* < 0.001, 95% Confidence Interval [CI]: 0.5610–0.8502]. All other Spearman’s rho data had large confidence intervals and did not permit definitive conclusions about the strength of the observed relationships. Spearman’s rho correlation analysis is summarized in Figure 8.

## 4. Discussion

This study demonstrated that a short program of mild exercise in cancer patients while receiving chemotherapy led to a reduction in state anxiety levels. Chemotherapy is an intense and cyclical treatment with many side effects, including hair loss, nausea, vomiting, and diarrhea. Prolonged treatment periods, repeated hospitalizations, and the side effects of chemotherapy, along with the awareness of having cancer, can significantly impact the psychological well-being of patients [27]. Furthermore, physical and cognitive impairments due to side effects of cancer treatment can significantly contribute to a greater fear of cancer progression [28]. Chemotherapy-induced fatigue and physical symptoms may remind patients of their cancer or be misinterpreted as signs of its possible relapse, leading to increased fear of recurrence [29]. In this context, it seems reasonable that many studies report that coping with the effects of chemotherapy can be stressful, frustrating, and traumatic. Consequently, patients undergoing chemotherapy are at a higher risk of experiencing psychological and emotional difficulties, such as sleep disturbance, depression, and anxiety [27,28,29,30,31]. Among negative emotions, anxiety is the one most experienced by cancer patients [27,31]; it is not just a normal reaction to a cancer diagnosis, but can also arise from treatment, fear of recurrence, or even after completing treatment due to fear of recurrence. It can manifest in four ways: situational anxiety, disease-related anxiety, treatment-related anxiety, and an exacerbation of pre-existing anxiety disorders [27].

Exercise has emerged as a promising supportive care strategy that can alleviate some of these adverse effects [32]. Many studies have shown that exercise may improve mood, increase treatment tolerance and enhance the immune system [12,33,34]. Furthermore, research consistently demonstrates that exercise can be highly effective in alleviating both acute and chronic side effects of cancer and chemotherapy [35]. The primary outcomes examined in these studies include cancer-related fatigue, depression, anxiety, sleep disturbance, cognitive function, self-esteem, nausea, cardiopulmonary function, muscular strength, flexibility, and body composition [36,37,38]. Despite these benefits, adherence to exercise guidelines among cancer patients remains low, largely due to treatment side effects, lack of guidance, and patient misconceptions [39]. A lack of information regarding exercise during treatment may lead to patients feeling unsure about what they are allowed and able to do [39].

Exercise as an effective nonpharmacological therapy in cancer patients includes both aerobic and resistance training. Aerobic exercise is characterized by low intensity, enabling prolonged periods of activity. It engages large muscle groups and is rhythmic in nature. Common forms of aerobic exercise include walking, running, cycling, and swimming. Resistance training regimens target improvement in muscle hypertrophy, bone mineral density, strength, functional mobility and body composition [36].

However, before recommending exercise as a non-pharmacological therapy in cancer patients, its feasibility and safety must be carefully considered. Thomas et al. investigated the safety of exercise during chemotherapy in a randomized crossover trial [40]. The study involved 10 adults aged 18 to 60 who were receiving non-vesicant chemotherapy agents. Participants engaged in 20 min of supervised low intensity cycling, during which no adverse events or treatment interferences were reported. Participants in the exercise group experienced significantly reduced boredom; however, there were no significant differences observed in other symptoms following the intervention. In the meta-analysis of 19 studies by Singh et al., assessing the safety, feasibility, and effects of exercise in individuals with colorectal cancer, the authors concluded that there was no difference in the risk of adverse events between the exercise intervention and usual care groups [41].

Given that cancer patients often face a significant burden of symptoms and are frequently considered frail, it is reasonable to question whether all cancer patients are fit to engage in exercise during chemotherapy and throughout the active phase of their illness. However, the recent review by Avancini et al., regarding physical activity recommendations for cancer patients, indicates that the majority of the assessed guidelines—eight out of eleven—support engaging in physical activity for the entire oncological population, across all stages of cancer treatment, both during and after therapy [42].

A related and equally important consideration involves the type and dosage of exercise recommended for cancer patients. Regarding the types of activities recommended, all guidelines advocate for a combination of aerobics and resistance training. Most guidelines suggest an average physical activity dosage of 150 min per week of moderate-intensity aerobic training [or 75 min of vigorous-intensity activity, which is considered equivalent] along with resistance training twice per week. However, detailed guidance regarding session duration, intensity measurement, and progression is often limited across all guidelines. Three recommendations provide more comprehensive instructions, suggesting at least three sessions per week of aerobic training lasting 20–30 min, at moderate intensity, as well as 6–10 sets of resistance exercise performed at least 1–3 times per week with 1–4 sets of 8–15 repetitions at moderate intensity [at least 50–60% of the one-maximal repetitions] [41,43,44].

The appropriate prescription of exercise for individuals living with and beyond cancer has yet to be clearly established, as there is still no consensus within the scientific community. Although the recently published exercise guidelines from the American College of Sports Medicine (ACSM) suggest that it is possible to tailor specific exercise prescriptions—such as frequency, intensity, time, and type of exercise—to improve various cancer-related health outcomes, the American Society of Clinical Oncology argues that current evidence is insufficient to provide specific exercise dosage recommendations [45].

This discrepancy in guidance likely contributes to the fact that many patients undergoing chemotherapy do not receive adequate information or individualized advice regarding exercise. Furthermore, most existing studies have focused on incorporating exercise protocols that patients can perform at home, either during or after the completion of chemotherapy regimens. Few studies have examined the feasibility of implementing exercise for patients with cancer directly within the chemotherapy infusion setting, under the supervision of a trained professional. Kerrigan et al. conducted a pilot study demonstrating that aerobic exercise during chemotherapy infusions is both safe and well tolerated [46]. In this study, ten breast cancer patients participating in the ExCITE [Exercise and Cancer Integrative Therapy and Education Program] trial at Henry Ford Hospital performed mild aerobic exercise on a portable leg ergometry machine [Monarck], while receiving chemotherapy. This activity was supplemented by a prescribed outpatient exercise program. During the infusion and simultaneous exercise, the patients’ heart rates were maintained at approximately 30–40% of heart rate reserve and all sessions were supervised by a clinical exercise physiologist. Across 55 chemotherapy infusions of those 10 patients, 18 sessions included exercise, while 37 did not, and no adverse events were reported.

The Exercise in All Chemotherapy trial further explored this model by employing an exercise professional directly into the chemotherapy infusion suite in order to include exercise as a standard part of cancer care [18]. The exercise regimens provided to patients were individualized based on the results of brief baseline functional testing. This study concluded that this approach is both acceptable and feasible from the perspective of clinicians and patients, indicating strong potential for integration into routine oncology care. The same conclusion was supported by Schmitz et al., who provided a comprehensive analysis of the ENACT trial, demonstrating that exercise during chemotherapy is both feasible and beneficial [18]. In their study, patients received detailed exercise prescriptions—including frequency, intensity, and duration—and were encouraged to perform independently at home, between chemotherapy visits.

In our study, we observed that a 20-minute program of gentle exercise and stretching conducted during chemotherapy sessions, tailored to each patients’ physical status, had a positive impact on state anxiety levels. The clinical significance of this finding is very important, as high levels of anxiety can trigger physiological responses mediated by the autonomic nervous system, resulting in increased pain perception, nausea, and vomiting—factors that can subsequently further intensify distress. Moreover, anxiety can induce a state of behavioral paralysis and thus hinder the patient’s ability to cope with chemotherapy-related side effects [30]. As a result, anxiety may adversely affect compliance with treatment protocols [47].

The systematic review and meta-analysis by Wang et al. of 51 cohort studies revealed that clinically diagnosed anxiety disorders and anxiety symptoms were associated with increased cancer-specific mortality as well as poorer overall survival [48]. Anxiety and distress are both negative emotional states, but they differ in their nature and intensity. Anxiety is characterized by an excessive sense of tension, worry or apprehension, relative to potential future threats [49]. In contrast, distress is a broader term encompassing various unpleasant or uncomfortable emotional states, which can include anxiety, depression and other negative feelings [50]. In this context, anxiety can be viewed as a specific manifestation of psychological distress.

Cancer-related distress is defined as “an unpleasant experience of psychological [cognitive, behavioral, emotional], physical, social, and/or spiritual nature that may interfere with the ability to cope effectively with cancer, its physical symptoms, and its treatment” [51]. This distress is multifactorial in cancer patients and often begins at the time of diagnosis. It tends to intensify over the course of the disease due to both the burden of cancer-related symptoms and toxicity associated with therapy [52].

The management of patients with cancer not only aims to achieve remission and enhance survival rates but also places significant emphasis on reducing psychological distress and enhancing patients’ QOL. This dual approach is essential, as an improved QOL fosters greater adherence to treatment protocols, encourages patients to complete therapy, and facilitates the effective management of symptoms [53].

In our study, we used the Spielberger State–Trait Anxiety Inventory [STAI] to assess anxiety levels, specifically focusing on the STAI-Y1 scale, which measures state anxiety. While both state and trait anxiety are important concepts, they serve different purposes in understanding and addressing anxiety. State anxiety reflects a temporary emotional condition triggered by specific situations or stressors [54], making it particularly useful for evaluating patients’ immediate psychological responses to cancer treatment [49]. It reflects a transient condition rather than a pathological state, and it can fluctuate rapidly depending on the situation and the individual’s coping capacity.

In contrast, trait anxiety represents a stable predisposition to experience anxiety across a range of situations and is more relevant for understanding long-term emotional patterns and their influence on overall well-being. Trait anxiety is considered a more stable aspect of personality, as it is considered to be part of a broader spectrum of personality traits, and has been linked to various psychological conditions [54]. It represents a consistent individual tendency of a person to respond with worry, concern and apprehension to various situations. Trait anxiety helps us understand individual variability in susceptibility to anxiety disorders and predict how individuals might react to stressful situations. It is relatively stable over time and across different situations. Individuals with higher trait anxiety often experience higher levels of state anxiety in response to stressors. While the two concepts are interrelated, there is research suggesting that trait and state anxiety may have partially distinct brain mapping [49]. This supports the value of assessing both to gain a comprehensive understanding of anxiety.

In our study, we chose to use only the STAI-Y1 [state anxiety] section of Spielberger’s State–Trait Anxiety Inventory to measure changes in state anxiety levels. This decision was based on the specific focus of our intervention: the acute anxiety experienced during chemotherapy sessions, often driven by fear of side effects and psychological distress. Additionally, limiting the time required to assess the STAI-Y1 helped in reducing participant burden and limiting dropout, as the shorter format requires less time to complete. The significant number of patients that refused to participate in a short program of gentle physical activity bears out the fact that (a) patients should be informed of current recommendations regarding exercise and (b) it should be explained to them that exercise is a vital and feasible component in the management of their health-related QOL [39].

### Limitations

This study has several limitations that should be acknowledged. First, the sample size was relatively small [n = 45]. While it met the minimum threshold for statistical power, the limited number of participants may affect the generalizability of the findings. Second, the study population was drawn exclusively from a single hospital located in the northern region of Athens, specifically from its Day Care Unit, which may introduce selection bias and limit the applicability of the findings to broader cancer populations. Third, the intervention consisted only of a single 20-minute session, and the absence of a follow-up period restricts our ability to assess the sustained or long-term effects of the intervention on anxiety levels. Additionally, the inclusion of patients with different cancer types and treatment stages, without stratified analysis, introduces heterogeneity that may have impacted the consistency and interpretability of the findings.

From a methodological standpoint, the absence of a control group limits our ability to attribute changes in anxiety solely to the intervention, as other contextual factors may have influenced outcomes. The reliance on self-reported anxiety level introduces potential bias, including the tendency for participants to provide responses they perceive as socially acceptable or desirable.

Lastly, a significant number of eligible patients declined participation—often due to concerns about fatigue or injury—raising the possibility of selection bias, as those who participated may have been more physically or psychologically predisposed to engage in the program. It is also important to note that in Greece patients undergoing chemotherapy are not typically offered exercise as part of their care and may be unaware of its feasibility and safety. This unfamiliarity likely contributed to the reluctance of many patients to consent to participation in the study.

## 5. Conclusions

This study investigated the effects of mild exercise during chemotherapy sessions on patients’ state anxiety levels. The findings indicate that brief, patient-tailored exercise can lead to a short-term reduction in anxiety. These results suggest that incorporating mild exercise during chemotherapy may help alleviate anxiety and psychological burden. However, these conclusions should be viewed with caution due to the aforementioned limitations of this study and its design. Further studies are warranted to determine the optimal type, intensity and duration of exercise across different types of cancer patients.

## Figures and Tables

**Table 1:** Program of mild exercise used in the study.

Exercise Stage	Included Exercises
I. Warm-Up [5 min]:	Seated ankle plantar flexion/dorsiflexion: 20 repetitions (Figure 1(I1b,I1c)).
	2. Seated knee extensions: 10 repetitions, holding for 5 s each (Figure 1(I-2a,I-2b)).
	3. Seated alternating knee lifts: 20 repetitions (Figure 1(I-3)).
II. Main Exercise [10 min]:	Seated torso rotations: 20 repetitions (Figure 2(II-1b,II-1c)).
	2. Hand gripping: 20 repetitions (Figure 2(II-2a,II-2b)).
	3. Forearm supination and forearm pronation: 10 repetitions, both arms (Figure 2(II-3a,II-3b)).
	4. Seated or standing elbow flexion: 15 repetitions (Figure 3(II-4a–II-4c)).
	5. Seated or standing shoulder flexion: 10 repetitions (Figure 3(II-5a–II-5c)).
	6. Seated or standing shoulder abduction: 10 repetitions (Figure 3(II-6a–II-6c)).
	7. Standing spot marches: 20 steps (Figure 4(II-7a–II-7c)).
	8. Standing knee-high marches: 10 repetitions (Figure 4(II-8a,II-8b)).
	9. Light lunges: 5 repetitions per leg [optional] (Figure 4(II-9a,II-9b)).
III. Cool-Down [5 min]:	Standing lateral torso bends: 5 repetitions on each side (Figure 5(III-1a–III-1c)).
	2. Standing lateral torso bends with arms raised: 5 repetitions on each side (Figure 5(III-2a–III-2c)).
	3. Overhead stretches: hold the stretch for 10 s (Figure 5(III-3)).
	4. Seated hamstring stretches: hold for 10 s. (Figure 5(III-4a,III-4b)).
	5. Deep breathing exercises: complete 3 cycles of deep breathing (Figure 5(III-5a–III-5c)).

## Data Availability

Data are available upon request from the first author.

## Supplementary Materials

The following supporting information can be downloaded at: https://www.mdpi.com/article/10.3390/jcm14155591/s1, Table S1: Patient data.

## Abbreviations

The following abbreviations are used in this manuscript:

QoL	Quality of Life
STAI	State–Trait Anxiety Inventory
CI	Confidence Interval
ACSM	American College of Sports Medicine
ExCITE	Exercise and Cancer Integrative Therapy and Education Program
