# Supplementary material for: The Effect of Mild Exercise in the Chemotherapy Room on the Anxiety Level of Cancer Patients: A Prospective Observational Paired Cohort Study
Source: J Clin Med. 2025 Aug 7;14(15):5591. doi: 10.3390/jcm14155591 (PMC12347337; doi:10.3390/jcm14155591)
Supplement: Supplementary file 1 [file jcm-14-05591-s001.zip › jcm-3757498-supplementary.pdf]

Patient ID	Gender	Age	STAI -Y1 pre-exercise intervention	STAI-Y1 post-exercise intervention
1	Female	74	39	34
2	Male	55	44	45
3	Female	77	42	47
4	Male	81	57	52
5	Male	76	21	20
6	Male	75	37	21
7	Male	78	31	21
8	Female	65	41	20
9	Female	59	42	41
10	Male	76	22	20
11	Female	55	26	21
12	Male	81	23	20
13	Male	60	31	32
14	Male	70	32	35
15	Female	83	30	21
16	Male	69	43	38
17	Female	83	46	21
18	Female	67	25	36
19	Male	68	42	38
20	Female	60	35	37
21	Female	53	51	30
22	Male	65	33	37
23	Female	64	45	24
24	Female	78	69	60
25	Female	77	29	41
26	Male	57	30	21
27	Female	46	65	49
28	Male	70	36	31
29	Male	81	58	37
30	Female	48	23	20
31	Male	78	33	21
32	Female	61	64	79
33	Female	60	47	35
34	Female	64	24	21
35	Female	49	42	30
36	Male	85	39	23
37	Male	68	29	22
38	Male	76	20	20
39	Male	73	37	32
40	Male	57	23	20
41	Female	83	25	20
42	Male	78	73	75

43	Male	75	30	22
44	Male	63	40	30
45	Male	85	24	20
